# High-Riding Conus Medullaris Syndrome: A Case Report and Literature Review—Its Comparison with Cauda Equina Syndrome

**DOI:** 10.3390/tomography9060156

**Published:** 2023-10-27

**Authors:** Ya-Lin Huang, Shin-Tsu Chang

**Affiliations:** 1 Department of Medical Education, Kaohsiung Veterans General Hospital, Kaohsiung 813414, Taiwan; yalinhuang99@gmail.com; 2 School of Medicine, National Defense Medical Center, Tri-Service General Hospital, Taipei 114201, Taiwan; 3 Department of Physical Medicine and Rehabilitation, Kaohsiung Veterans General Hospital, Kaohsiung 813414, Taiwan

**Keywords:** conus medullaris syndrome, upper motor neuron sign, burst fracture, SPECT, case report

## Abstract

Introduction: Conus medullaris syndrome (CMS) is a distinctive spinal cord injury (SCI), which presents with varying degrees of upper motor neuron signs (UMNS) and lower motor neuron signs (LMNS). Herein, we present a case with a burst fracture injury at the proximal Conus Medullaris (CM). Case Presentation: A 48-year-old Taiwanese male presenting with lower back pain and paraparesis was having difficulty standing independently after a traumatic fall. An Imaging survey showed an incomplete D burst fracture of the T12 vertebra. Posterior decompression surgery was subsequently performed. However, spasticity and back pain persisted for four months after surgical intervention. Follow-up imaging with single photon emission computed tomography (SPECT) and a whole body bone scan both showed an increased uptake in the T12 vertebra. Conclusion: The high-riding injury site for CMS is related to a more exclusive clinical representation of UMNS. Our case’s persistent UMNS and scintigraphy findings during follow-up showcase the prolonged recovery period of a UMN injury. In conclusion, our study provides a different perspective on approaching follow-up for CM injuries, namely using scientigraphy techniques to confirm localization of persistent injury during the course of post-operative rehabilitation. Furthermore, we also offered a new technique for analyzing the location of lumbosacral injuries, and that is to measure the location of the injury relative to the tip of the CM. This, along with clinical neurological examination, assesses the extent to which the UMN is involved in patients with CMS, and is possibly a notable predictive tool for clinicians for the regeneration time frame and functional outcome of patients with lumbosacral injuries in the future.

## 1. Introduction

Conus Medullaris Syndrome (CMS) is a severe medical emergency, namely, it is known to significantly hinder one’s ability to perform activities of daily living and furthermore cause long-term impairment if left untreated [1,2]. CMS occurs when the conus medullaris (CM), a cone-shaped structure tapered at the end of the lumbar enlargement of the spinal cord and terminating around L1–L2 [3], is compressed. The filum-like structures just below the conus medullaris are named the cauda equina (CE), and compressions of the CE are called Cauda Equina Syndrome (CES) [4,5].

CMS and CES are often compared and contrasted in the literature due to the proximity of their anatomical positions and their distinctive clinical representations. Due to its Central Nervous System (CNS) involvement, CMS often exhibits upper motor neuron signs (UMNS) such as spasticity, while CES presents lower neuron signs (LMNS) such as flaccidity [4,5,6]. According to the literature, CMS is likely to exhibit a mixture of both UMNS and LMNS due to its anatomical composition; having nerve roots directly attached to it means that the lower motor neurons will be affected as well [2,6,7,8].

Single Emission Computer Tomography (SPECT) is a radiographic technique routinely used in oncology and neurology due to its remarkable sensitivity in detecting inflammation. SPECT relies on the administration of radiotracers, specifically, in our case, Technetrium-99m methylene diphosphonate (Tc-99m MDP). Detection of the increased uptake of radiotracers relies on high-energy photons that arise from the interaction between the Tc-99m MDP radiotracers and amorphous calcium phosphate within areas of increased osteogenic activity such as inflammation and metastasis.

The purpose of this case report is (1) to present a case of CMS with a more proximal than average site of injury superior to the tip of CM, (2) to discuss the clinical findings in comparison with average injury site of CM as well as with CES, and (3) to assess the outcome after surgical decompression using scintigraphy analysis.

## 2. Case Report

### 2.1. History

A 48-year-old Taiwanese male with a medical history of previous epidural hematoma (EDH), hypertension, and a personal history of alcohol addiction (up to 700 mL of whisky per day for 10 years), betel nut chewing, and cigarette smoking (2 packs per day) was brought to the ER after falling posteriorly during a conscious disturbance episode. The patient had been withdrawing from alcohol for two days.

### 2.2. Examination

Initial physical examination found a wound over the lower limb and right elbow, laboratory tests found elevated liver enzymes, lactate, and leukocytosis. Brain Computed Tomography (CT) revealed the presence of a hypodense area with brain tissue loss in the right temporal region, suspected to be a previous brain insult. Under the effect of alcohol withdrawal syndrome, the patient was admitted to the neurology ward for further management. Initial treatments included intravenous Lorazepam 0.5-amp Q8H for alcohol withdrawal syndrome and oral Levetiracetam 500 mg BID for seizure control.

The patient regained consciousness 14 h after admission, however, new findings were quickly brought to our attention: lower back pain and paraparesis, due to which the patient was having difficulty standing independently; these symptoms were accompanied by urinary retention and bilateral saddle anesthesia. There was no significant change in Deep Tendon Reflex (DTR) or spasticity, suspect to be a spinal shock related finding. Muscle power was tested to be Right Upper Limb (RUL)(5)/Right Lower Limb (RLL) (5)/Left Upper Limb (LUL) (4−)/Left Lower Limb (LLL) (4−); the last spinal level with full muscle power of 5 was at T1, which marked the motor level of injury at T1. There was slight impairment in light touch and pin prick sensations below T12, a score of 2 bilaterally above T12 and a 1 bilaterally below T12, respectively, which marked the sensory level of injury at T12. Deep anal pressure and voluntary anal contraction were both present. Analyzing both the sensory level and motor level of injury, the final neurological level of injury was determined to be at T12. The fracture was classified as Incomplete D on the American Spinal Cord Injury Association’s International Standards for Neurological Classification of Spinal Cord Injury (ISNSCI) and type A3 injury according to the AO Spine Subaxial Classification System.

**Figure 1:**
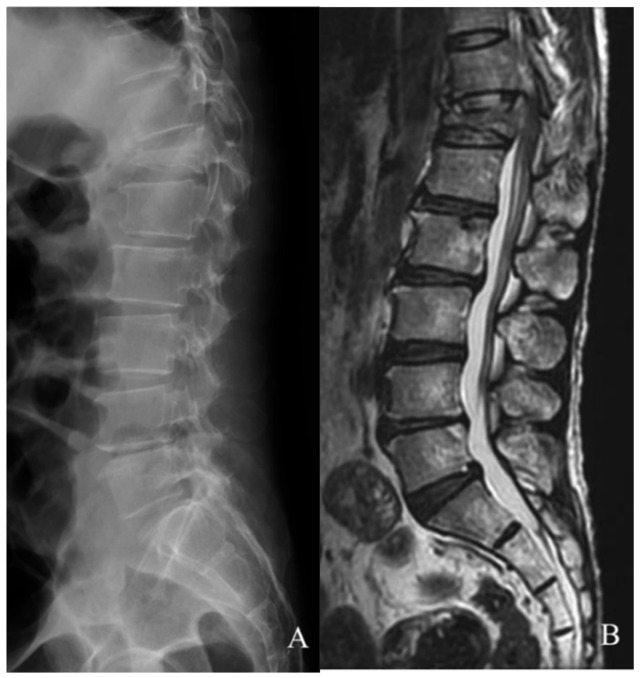
Preoperative imaging (**A**,**B**). A burst fracture at T12 resulted in a pan-shaped fragment compressing the conus medullaris that was revealed via X-ray (**A**) and T2-weighted MRI (**B**), respectively.

Imaging studies were ordered to survey spinal involvement: the X-ray results showed a T12 compression injury (Figure 1A), then MRI confirmed a recent burst fracture of the T12 vertebra with severe collapse of the middle column, resulting in compression of the anterior aural sac containing proximal CM by retroposed bone fragments (Figure 1B). 

### 2.3. Surgery and Post-Operative Findings

**Figure 2:**
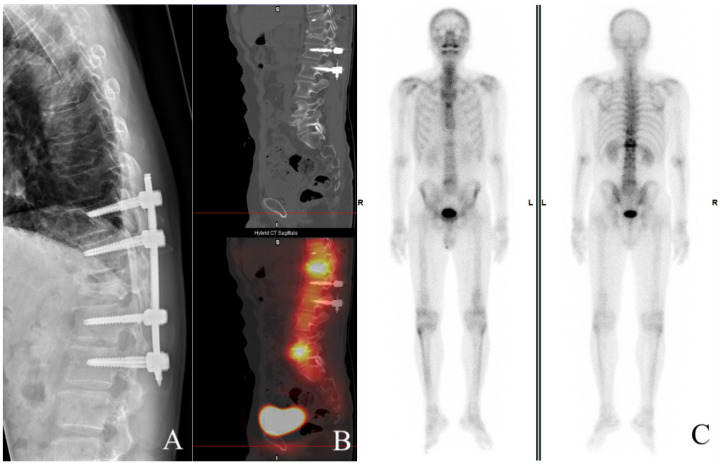
Postoperative follow-up at 4 months (**A**–**C**). X-ray documents transpedicular screw fixation of T10,11-L1,2 with posterior-lateral fusion. Obvious compression of T12 appears (**A**) and increased uptake, is shown on both SPECT (**B**) and whole body bone scan (**C**), respectively.

Surgical intervention was scheduled 48 h after admission; the patient underwent posterior decompression via T1–12 laminectomy and transpedicular screw fixation of T10,11–L1,2 with posterior-lateral fusion (Figure 2A). The patient regained urinary continence and was able to walk independently with a quad cane during his 4-month follow-up visit post-operation, slow gait was observed due to minimal heel strike distance from the previous stance; he wore a thoracolumbosacral orthosis for stabilization.

However, abnormal clinical findings were still present, according to the patient himself, there was still lower back pain, saddle anesthesia, and lower limb numbness. Neurological examination found a DTR increase in the bilateral lower limbs with RUL (2+)/RLL (4+)/LUL (2+)/LLL (4+), and Babinski’s sign was present bilaterally. The patient was alert and oriented E4V5M6, and speech was fluent, capable of comprehension and repetition. No abnormalities were found in the cranial nerve examinations. There was a slight decrease in bilateral lower limb muscle power, RUL (5)/RLL (4)/LUL (5)/LLL (4), and sensory impairment in light touch and pin prick sensations were still present below T12. Spasticity was apparent on bilateral lower limbs, RUL (0)/RLL (2)/LUL (0)/LLL (2) on the modified Ashworth scale.

Increased uptake on follow-up imaging studies correlated with our clinical findings, in which both Single Photon Emission Computed Tomography (SPECT) (Figure 2B) and a whole body bone scan (Figure 2C) showed increased uptake in the mid- to anterior column. He is currently undertaking acupuncture, physical therapy, occupational therapy, and rehabilitation in the hope of regaining more independence in performing activities of daily living.

## 3. Discussion

Our case was diagnosed with an incomplete D burst fracture injury at the T12 vertebra compressing proximal CM after a traumatic fall. The high-riding position of the injury is key to explaining the delayed neurological recovery indicated by persistent UMNS and SPECT findings.

**Figure 3:**
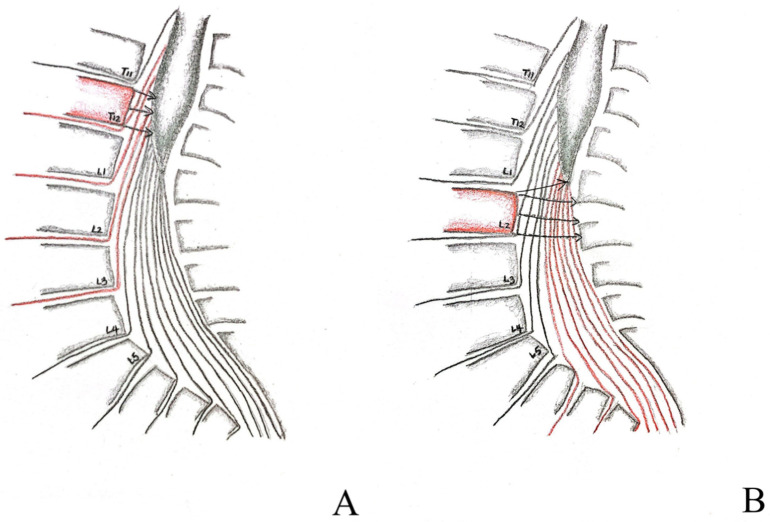
Graphical depiction of proximal and distal injuries to the conus medullaris (**A**,**B**). Proximal injuries relative to the tip of conus medullaris, as depicted by the T12 vertebra shaded in red, affects predominately UMN roots, depicted by the arrowheads compressing the conus medullaris, resembles the condition of our case (**A**) as opposed to distal injury, depicted by the L2 vertebra shaded in red, which affects more LMN roots, depicted by the arrowheads compressing not only the tip of the conus medullaris but also the cauda equina extending underneath, this will result in a more similar clinical presentation to that of cauda equina syndrome (**B**).

CMS is vague by definition because the range of the anatomical location of CM varies from person to person, from the lower third of T11 to the upper third of L3; because of this variation, the definition of CMS also varies within different studies. A systemic review by Brouwers and colleagues in 2017 compiled 1046 articles focused on patients with traumatic CMS and/or CES [1], defining the location of CMS as an injury of the vertebrae T12–L2. Furthermore, the injury associated with it includes structures from the spinal cord segment at T12 to the nerve root S5. Our case is cohesive with this definition and was positioned at the top of the spectrum. Because the natural anatomical location of CM is variable, we suggest when analyzing CMS injuries not only looking for the site of injury but also its location relative to the tip of the CM (Figure 3A,B); injury below the tip of the CM is considered CES. The CES is defined as injury of vertebrae L3–L5, and is associated with damage to nerve roots L3–L5. Our imaging findings from MRI saw the tip of the CM in the upper third of L1, well below the location of compression, thus we excluded the possibility of CES. Concerning the entity contributing to the cord compression, it is either a vertebra, like in our case, a retroposed bone fragment after the burst fracture of the T12 vertebra, or a protruded disc, a popular etiology. We have presented a case with CES attributed to a bony fragment of L1 with a pan-shaped pattern recently [9]. This is the second case to identify the entity as a vertebral fragment causing such a disastrous situation. Additional factors, such as neoplasm, can attribute to either CES [10] or CMS [11] as well and should be distinguished carefully.

As a component of the spinal cord, the CM is associated with a group of nerve fibers called the Upper Motor Neurons (UMN), these are fibers from the central nervous system (CNS) that carry out voluntary movement, with the pyramidal tract being their main component. The pyramidal tract is further divided into the corticospinal tract, which synapses with the spinal cord nerve fibers and carries out the movement of the limbs; and the corticobulbar tract, which synapses with the cranial nerves and carries out the movement of the face [7]. Damage to these tracts is called a UMN lesion and these lead to what is called Upper Motor Neuron Signs (UMNS). The clinical presentation of UMNS includes weakness, spasticity, and hyperreflexia. What is noteworthy is that the weakness is usually associated with extensors of the upper limb and flexors of the lower limb; our case corresponds to this feature through the slow gait presented during follow-up because the flexors of the lower limb are responsible for the swing phase of the gait [12]. Weak flexors lead to decreased room for acceleration for the next heel strike and consequently, less leverage to propel forward in. Our case exhibits mainly UMNS both before and after operation. Other symptoms such as flaccidity are associated with another phenomenon called Lower Motor Neuron Signs (LMNS). Lower Motor Neurons (LMN) can originate from both the CNS and PNS and synapse directly with muscles [8], hence both CMS and CES can exhibit similar LMNS features (Table 1).

It is not by coincidence that the UMNS are present longer than the LMNS in our case, even after decompression surgery [13,14,15]. A meta-analysis by Khorasanizadel and colleagues included a total of 114 studies reporting American Spinal Injury Association Impairment Scale (AIS)/Frankel classification changes in 19,913 patients. The study divided patients into four groups of SCI: cervical (C1–T1), thoracic (T2–T10), thoracolumbar (T11–L2), and lumbar/cauda equina (L3 and lower), respectively, each group was then further divided into four levels of AIS/Frankel subgroups. Injury in the lumbar region showed a significantly better prognosis than any other levels of injury, specifically, it had a higher proportion of patients whose AIS/Frankel scoring improved by at least one grade, in all initial AIS/Frankel grading categories [16]. Similarly, according to Harrop and colleagues [8], injury to T4–T9 had fewer neurological improvements than T10–T12, suspected to be due to the additional lower neurons present in T10–T12. The UMN regenerates slower than the LMN, and therefore the functional outcome of CMS could be relatively worse than CES [17,18,19,20,21,22]. Our imaging findings, namely the obvious increase of uptake for SPECT at the T12, confirmed the localization of persistent injury, which is likely to have contributed to the presence of lower back pain and UMNS during our patient’s 4 month post-operative follow-up.

**Table 1:** Summarized comparison of Conus Medullaris Syndrome (CMS) with Cauda Equina Syndrome (CES) from the literature review.

	CMS	CES	References
Vertebrae damaged	T12–L2	L3–L5	[1,7,8]
Nerve roots damaged	T12–S5	L3–L5	[1,7,8]
Composition	UMN predominant (depending on location relative to tip of conus)	LMN only	[7,8]
Muscle tone	Spastic (Prominent in upper limb flexors and lower limb extensors)	Flaccid	[7,8]
Stretch (tendon) reflex	Increased	Decreased	[7]
Weakness	UMN type (Prominent in upper limb extensors and lower limb flexors with diminished maximal discharge frequency on EMG)	LMN type (Delayed or reduced recruitment of motor units on EMG)	[7,22]
Lower back pain	Present	Present	[18,19,20,21]
Symmetry of weakness in lower extremities	Symmetric	Asymmetric	[18,19,20,21]
Saddle anesthesia (S3–S5)	Symmetric	Asymmetric	[18,19,20,21]
Bladder and rectal dysfunction	LMN type (Atonic bladder and flaccid anal sphincter)	Relative sparing	[16,22]
Regeneration time frame	Slow	Relatively faster	[17,18,19,20,21]
Functional outcome	Worse	Relatively better	[17,18,19,20,21]

There are some major limitations to this study, however. First is the sample size; we analyzed only one patient in this study, and our method of analysis may be different from other studies. Furthermore, the location of the upper border of the CM relative to the tip is yet to be defined, and might result in variations from past studies due to the possible inclusion of lumbar spine injuries that are not CMS. Finally, there was no endpoint set for this study and the patient may regain more function with time, which may produce different interpretations of the results.

## 4. Conclusions

The high-riding injury site for CMS is related to a more exclusive clinical representation of UMNS. Our case’s persistent UMNS and scintigraphy findings during follow-up showcase the prolonged recovery period of UMN compared to LMN. In conclusion, our study provides a different perspective on approaching follow-up for CM injuries, namely using imaging techniques to confirm localization of persistent injury during the course of post-operative rehabilitation. Furthermore, we also offered a new technique for analyzing the location of lumbosacral injuries, and that is to measure the location of the injury relative to the tip of the CM. This, along with clinical neurological examination, assesses the extent to which the UMN is involved in patients with CMS, and is possibly a notable predictive tool for clinicians for the regeneration time frame and functional outcome of patients with lumbosacral injury in the future.

## Data Availability

Data will be made available on request.

## References

[1] Brouwers E., van de Meent H., Curt A., Starremans B., Hosman A., Bartels R. (2017). Definitions of traumatic conus medullaris and cauda equina syndrome: A systematic literature review. Spinal Cord.

[2] Chen J., Prasanna K., Tan Y.L. (2022). The ambulatory outcomes of traumatic cauda equina syndrome and conus medullaris syndrome: A systematic review. Arch. Phys. Med. Rehabil..

[3] Nakashima H., Ito K., Katayama Y., Tsushima M., Ando K., Kobayashi K., Machino M., Ito S., Koshimizu H., Segi N. (2021). The Level of Conus Medullaris in 629 Healthy Japanese Individuals. J. Clin. Med..

[4] Bulloch L., Thompson K., Spector L. (2022). Cauda Equina Syndrome. Orthop. Clin. N. Am..

[5] Woodfield J., Hoeritzauer I., Jamjoom A.A., Jung J., Lammy S., Pronin S., Watts A., Hughes L., Moon R.D., Darwish S. (2023). Presentation, management, and outcomes of cauda equina syndrome up to one year after surgery, using clinician and participant reporting: A multi-centre prospective cohort study. Lancet Reg. Health Eur..

[6] Quaile A. (2019). Cauda equina syndrome—The questions. Int. Orthop..

[7] Esquenazi A. (2009). Upper motor neurone syndrome and spasticity. Lancet Neurol..

[8] Garg N., Park S.B., Vucic S., Yiannikas C., Spies J., Howells J., Huynh W., Matamala J.M., Krishnan A.V., Pollard J.D. (2017). Differentiating lower motor neuron syndromes. J. Neurol. Neurosurg. Psychiatry.

[9] Hong Y.-H., Wang G.-J., Lan H.H.-C., Chang S.-T. (2019). Cauda equina compressed by bratpfannen-like vertebra rather than protruded disc: An unusual radiographic images in a lady with severe osteoporosis. Mathews J. Case Rep..

[10] Chang C.C., Chang S.T. (2008). Dual malignant peripheral nerve sheath tumors: Common in nature but different in images. Eur. Neurol..

[11] Hsu K.C., Li T.Y., Chu H.Y., Chen L.C., Chang S.T., Wu Y.T. (2013). Conus medullaris metastasis in breast cancer: Report of a case and a review of the literature. Surg. Today.

[12] Ravera E.P., Catalfamo P.A., Crespo M.J., Braidot A.A.A. (2012). Electromyography as an important parameter for a proper assessment of dynamic muscles strength in gait analysis. Am. J. Biomed. Eng..

[13] Kingwell S.P., Curt A., Dvorak M.F. (2008). Factors affecting neurological outcome in traumatic conus medullaris and cauda equina injuries. Neurosurg. Focus.

[14] Hashimoto T., Kaneda K., Abumi K. (1988). Relationship between traumatic spinal canal stenosis and neurologic deficits in thoracolumbar burst fractures. Spine.

[15] Park S.E., Elliott S., Noonan V.K., Thorogood N.P., Fallah N., Aludino A., Dvorak M.F. (2017). Impact of bladder, bowel and sexual dysfunction on health status of people with thoracolumbar spinal cord injuries living in the community. J. Spinal Cord Med..

[16] Khorasanizadeh M., Yousefifard M., Eskian M., Lu Y., Chalangari M., Harrop J.S., Jazayeri S.B., Seyedpour S., Khodaei B., Hosseini M. (2019). Neurological recovery following traumatic spinal cord injury: A systematic review and meta-analysis. J. Neurosurg. Spine.

[17] Tator C.H. (1998). Biology of neurological recovery and functional restoration after spinal cord injury. Neurosurgery.

[18] Harrop J.S., Naroji S., Gil Maltenfort M., Ratliff J.K., Tjoumakaris S.I., Frank B., Anderson D.G., Albert T., Vaccaro A.R. (2011). Neurologic improvement after thoracic, thoracolumbar, and lumbar spinal cord (conus medullaris) injuries. Spine.

[19] Transfeldt E.E., White D., Bradford D.S., Roche B.R. (1990). Delayed anterior decompression in patients with spinal cord and cauda equina injuries of the thoracolumbar spine. Spine.

[20] Clohisy J.C., Akbarnia B.A., Bucholz R.D., Burkus J.K., Backer R.J. (1992). Neurologic Recovery Associated with Anterior Decompression of Spine Fractures at the thoracolumbar junction (T12–L1). Spine.

[21] Attabib N., Kurban D., Cheng C.L., Rivers C.S., Bailey C.S., Christie S., Ethans K., Flett H., Furlan J.C., Tsai E.C. (2021). Factors associated with recovery in motor strength, walking ability, and bowel and bladder function after traumatic cauda equina injury. J. Neurotrauma.

[22] Loscalzo J., Fauci A., Kasper D., Hauser S., Longo D., Jameson J.L. (2022). Disease of the Spinal Cord. Harrison’s Principles of Internal Medicine.

